# Association between the level of education and knowledge, attitudes and practices regarding dengue in the Caribbean region of Colombia

**DOI:** 10.1186/s12889-018-5055-z

**Published:** 2018-01-16

**Authors:** Fredi Alexander Diaz-Quijano, Ruth Aralí Martínez-Vega, Alfonso J. Rodriguez-Morales, Ronald Alexander Rojas-Calero, María Lucrecia Luna-González, Ronald Giovanny Díaz-Quijano

**Affiliations:** 1 Departamento de Epidemiologia, Faculdade de Saúde Pública, Universidade de São Paulo, São Paulo, Brazil; 2 Organización Latinoamericana para el Fomento de la Investigación en Salud [Latin American Organization for the Promotion of Research in Health], Bucaramanga, Santander Colombia; 3 Public Health and Infection Research Group, Faculty of Health Sciences, Universidad Tecnológica de Pereira, Pereira, Risaralda, Colombia

**Keywords:** Dengue, Knowledge, Attitudes, Practices, Education, Colombia

## Abstract

**Background:**

Community integration in dengue control requires assessments of knowledge, attitudes and practices (KAPs), which can vary widely according to demographic and educational factors. We aimed to describe and compare the KAPs according to level of education in municipalities in the Caribbean region of Colombia.

**Methods:**

A survey was administered from October to December 2015, including families selected through probabilistic sampling in eleven municipalities. The analysis focused on the comparative description of the responses according to level of education. The KAP prevalence ratios (PR) according to education were estimated using Poisson regression (robust), including age and sex as adjustment variables.

**Results:**

Out of 1057 participants, 1054 (99.7%) surveys were available for analysis, including 614 (58.3%) who had a high school level of education or higher and 440 (41.7%) who had a lower level of education (not high school graduates). The high school graduates showed a higher frequency of correct answers in relation to knowledge about dengue symptoms and transmission.

On the other hand, graduates showed a higher probability of practices and attitudes that favor dengue control, including not storing water in containers (PR: 2.2; 95% Confidence Interval [CI]: 1.42–3.43), attend community meetings (PR: 1.33; 95% CI: 1.07–1.65), educate family members and neighbors in prevention measures (PR: 1.35; 95% CI: 1.15–1.59).

**Conclusions:**

Level of education could be a key determinant of knowledge of the disease and its transmission, as well as attitudes and practices, especially those that involve the integration of community efforts for dengue control.

## Background

Dengue fever is the viral illness transmitted by arthropods with the highest incidence in the world and is a growing cause of mortality in the countries of Latin America and the Caribbean [1, 2]. In 2016, Colombia was the Latin American country with the highest dengue mortality rate (4 deaths per million inhabitants), contributing 199 of the 1032 cases of the continent [1].

Community participation in the activities for the control and early recognition of the disease is essential to reduce the burden associated with the incidence and mortality from dengue fever [3]. Therefore, community integration requires assessments of knowledge, attitudes and practices (KAPs). However, KAPs regarding dengue vary widely among endemic regions and countries [4–9]. This can condition the ability of a community to identify, treat and control these emerging and re-emerging diseases.

The Caribbean region of Colombia has shown a rising trend in the incidence of dengue over the last decade. In particular, over the last decade, the department of La Guajira has exceeded the national average, reaching an incidence higher than 200 cases per 100,000 inhabitants in 2013 [10]. This has made it particularly necessary to establish prevention and control measures involving the community in this region. Despite all of this, little is known about the KAPs regarding dengue in this region, which are of great importance in this context.

In addition to the above, it is essential to recognize the determinants of the KAPs in order to guide control strategies [11]. In this sense, level of education could be an essential factor for both the acquisition of knowledge, as well as the implementation of preventive measures. Therefore, the objective of this study is to describe the KAPs in municipalities of the Caribbean region of Colombia and to establish their association with participants’ level of education.

## Methods

**Fig. 1:**
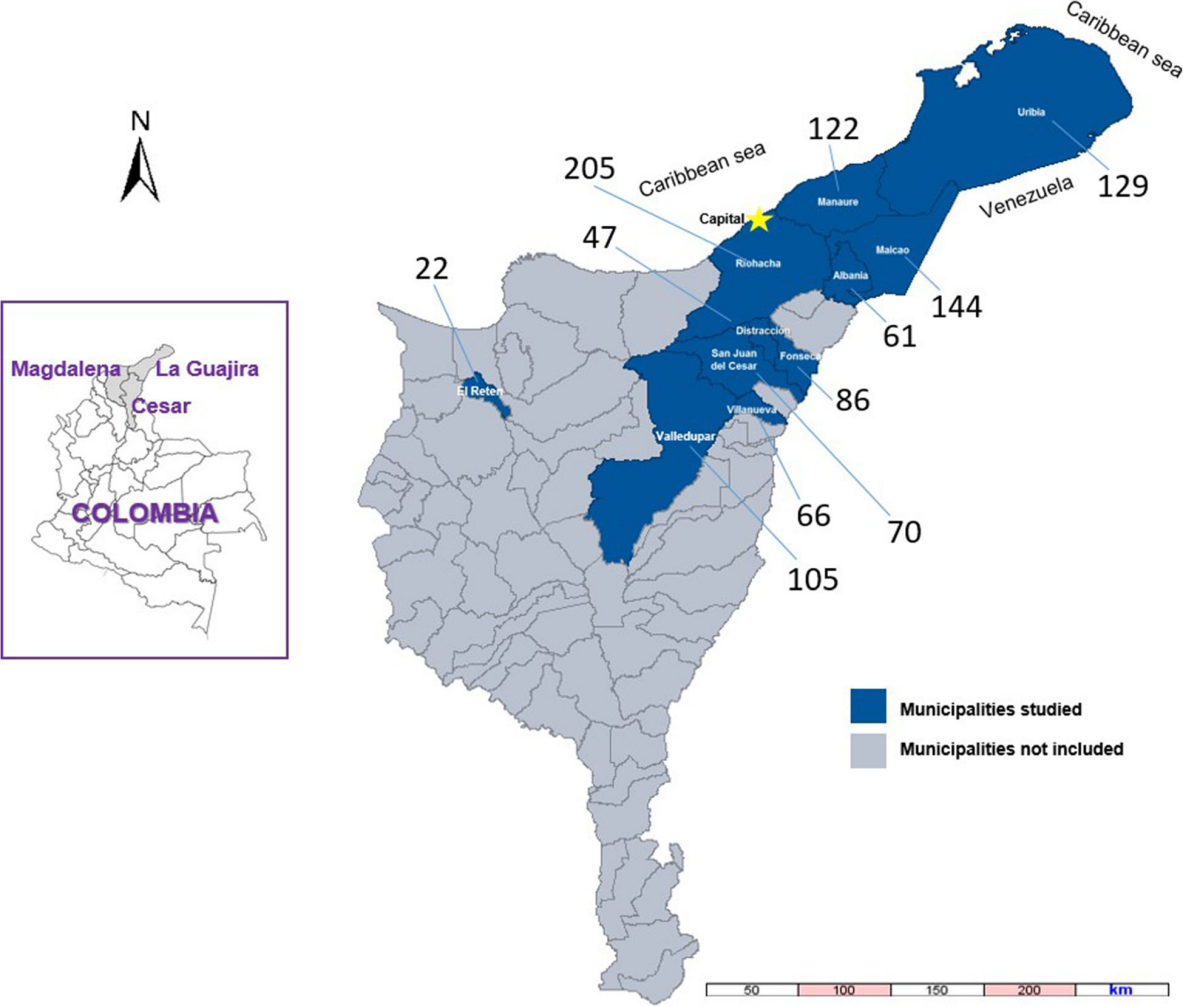
Distribution map of the number of surveys administered in participating municipalities. Departments of La Guajira, Cesar and Magdalena, Colombia

This paper is part of a population-based cohort study [10]. A survey was administered from October to December 2015, including families selected through probabilistic sampling by conglomerates in municipalities of 3 departments in Colombia: La Guajira, Cesar and Magdalena (Fig. 1). One adult from each family was interviewed by administering a structured questionnaire on KAPs regarding dengue. The form was based on one previously used by Cáceres et al. in another Colombian population [7].

Most of the surveys were administered in the Department of La Guajira (88%), which is located in the northern region of Colombia in which approximately 45% of the population is self-recognized as indigenous and nearly 15% as Afro-descendant. According to the census conducted in 2005, 32.1% of the population living in La Guajira has no education and 27.6% has only reached the basic primary level [12].

For this study, a sample was planned of at least 1000 families selected in 11 municipalities in the Colombian Caribbean (Fig. 1). The municipalities included nine in the department of La Guajira (Riohacha, Albania, Fonseca, San Juan del Cesar, Distracción, Maicao, Villanueva, Uribia and Manaure) which cover 87.2% of the population of this department [13]. Moreover, it included one municipality in Cesar (Valledupar) and another in Magdalena (El Retén).

The overall population living in the eleven municipalities was estimated at approximately 1.3 million inhabitants [13]. All of these municipalities were considered endemic for dengue, including three that were classified by the Ministry of Health and Social Protection as hyperendemic (Riohacha, Villanueva and Valledupar), seven as meso-endemic and one considered hypoendemic (El Retén) [14]. The number of homes per municipality was assigned considering the size of the population (Fig. 1), ranging from 22 families in El Retén (Magdalena) to 205 in Riohacha (La Guajira). Sampling by conglomerates was carried out for the selection of participants. Thus, blocks were randomly selected in each municipality and on each block, following a mapping and population analysis, a number of homes was randomly selected. The number of blocks selected per municipality was calculated in order to require the inclusion of between five and seven houses per block to reach the number of families assigned to the corresponding municipality. In each of the homes, residents were invited to participate and the questionnaire of this study was administered to the responsible adult who was at home at the time of the visit. When a family did not consent to participate, another house on the same block was randomly selected [10].

Interviews were applied by previously trained professional nurses, who carried out the informed consent process and the interview using a standardized questionnaire. This questionnaire included questions about KAPs regarding dengue disease and vector control. Each of the forms was typed in duplicate and the copies were compared. Typing inconsistencies were resolved by reviewing the original form.

### Statistical analysis

The analysis focused on the comparative description of the responses according to level of education. To this end, respondents who completed high school studies were compared to those with lower levels of education. For these comparisons, the Chi square test was used for categorical variables and the Mann-Whitney test was used for quantitative variables.

The associations identified between level of education and each of the responses regarding KAPs, were evaluated in a multiple model including age and sex as adjustment variables. This was done to estimate the prevalence ratio (PR) using the robust Poisson regression model for calculating the 95% confidence intervals (95% CI) [15]. Statistical significance was defined as a *p* value < 0.05 (two-tailed). The STATA v11.0 program was used for the statistical analysis.

## Results

A total of 1057 surveys was obtained, distributed in the 11 municipalities (Fig. 1), one of the surveys from the city of Manaure was excluded because it did not include data on age. In addition, two participants did not provide information on their level of education. 614 of the 1054 remaining respondents had a high school level of education or higher and 440 had a lower level of education (not high school graduates).

Most of the participants were female (81.3%) and the median age was 42 years (Table 1). Although the distribution by sex was not different in relation to the level of education, participants who were high school graduates were significantly younger compared to those who were not high school graduates (median age 37 years vs. 51 years, respectively [*p* < 0.001]). In relation to the disease, high school graduates more often recognized fever (98.4% vs 93.4%, p < 0.001), aches and pains (36.5% vs 29.2, *p* = 0.01) and petechiae (red dots on the skin: 13.4% vs 7.7%, *p* = 0.004) as signs of dengue. All other manifestations evaluated were recognized with similar frequency in the comparison groups (Table 1).

**Table 1:** Knowledge and practices in relation to the dengue disease and knowledge of its transmission according to level of education

Dependent variables	Total	High school graduates	Non-high school graduates	*P* value*
Female sex - No (%)	857/1054 (81.3%)	497/614 (80.9%)	360/440 (81.8%)	0.72
Age - Median (RIC)	42 (32–55)	37 (29–48)	51 (38–62)	< 0.001
Symptoms recognized as signs of dengue:				
Fever	1014/1053 (96.3%)	604/614 (98.4%)	410/439 (93.4%)	< 0.001
Aches and pains	352/1053 (33.4%)	224/614 (36.5%)	128/439 (29.2%)	0.01
Headache	474/1053 (45%)	277/614 (45.1%)	197/439 (44.9%)	0.94
Bone pain	282/1053 (26.8%)	168/614 (27.4%)	114/439 (26%)	0.61
Pain in the eyes	71/1053 (6.7%)	40/614 (6.5%)	31/439 (7.1%)	0.73
Sweating	51/1053 (4.8%)	33/614 (5.4%)	18/439 (4.1%)	0.34
Vomiting	471/1053 (44.7%)	283/614 (46.1%)	188/439 (42.8%)	0.29
Diarrhea	412/1053 (39.1%)	232/614 (37.8%)	180/439 (41%)	0.29
Stomach ache	79/1053 (7.5%)	43/614 (7%)	36/439 (8.2%)	0.47
Rash	94/1053 (8.9%)	61/614 (9.9%)	33/439 (7.5%)	0.17
Red spots on the skin	116/1053 (11%)	82/614 (13.4%)	34/439 (7.7%)	0.004
Bleeding gums	127/1053 (12.1%)	80/614 (13%)	47/439 (10.7%)	0.25
Nosebleed	138/1053 (13.1%)	88/614 (14.3%)	50/439 (11.4%)	0.16
Conduct when a family member has dengue:				
Care at home	26/1053 (2.5%)	15/614 (2.4%)	11/439 (2.5%)	0.95
Go to the doctor	553/1053 (52.5%)	317/614 (51.6%)	236/439 (53.8%)	0.49
Go to a health institution	29/1053 (2.8%)	310/614 (50.5%)	231/439 (52.6%)	0.50
Go to a pharmacy	1/1053 (0.1%)	0/614 (0%)	1/439 (0.2%)	0.24
Self-medicate	28/1053 (2.7%)	18/614 (2.9%)	10/439 (2.3%)	0.52
In the last month, someone in this family, living at home, at dengue:	28/1048 (2.7%)	17/610 (2.8%)	11/438 (2.5%)	0.79
Knowledge regarding transmission:				
Knows how dengue is transmitted	918/1051 (87.3%)	571/613 (93.1%)	347/438 (79.2%)	< 0.001
Knows the name of the mosquito	266/1051 (25.3%)	202/613 (33%)	64/438 (14.6%)	< 0.001
Knows what the mosquito looks like	395/1052 (37.5%)	266/614 (43.3%)	129/438 (29.5%)	< 0.001
Recognizes mosquito larvae	853/1050 (81.2%)	504/614 (82.1%)	349/436 (80%)	0.40
Means where the mosquito is believed to reproduce				
Stagnant water	518/1052 (49.2%)	302/613 (49.3%)	216/439 (49.2%)	0.98
Clean water	438/1052 (41.6%)	276/613 (45%)	162/439 (36.9%)	0.01
Rainwater	57/1052 (5.4%)	25/613 (4.1%)	32/439 (7.3%)	0.02
Dirty water	194/1052 (18.4%)	104/613 (17%)	90/439 (20.5%)	0.14
Drain water	41/1052 (3.9%)	19/613 (3.1%)	22/439 (5%)	0.11

*p-value was obtained by using the Chi square test for categorical variables. For the age variable, the p-value was obtained using the Mann-Whitney test

In relation to behavior, most respondents indicated that they would turn to a health institution or doctor when a family member had an illness compatible with dengue. In addition, 28 respondents stated that they had a family member, who lived in the same house, who got dengue in the last month. These responses on the behavior and the frequency of illness in the family had a similar frequency between high school graduates and non-graduates (*p* ≥ 0.50; Table 1).

In relation to knowledge about transmission, 87.4% of respondents knew how the disease was transmitted, 25.4% knew the name of the mosquito and 37.6% knew what the mosquito looked like. This knowledge was significantly more frequent in high school graduates compared with those with lower levels of education (*p* < 0.001; Table 1). In turn, high school graduates more frequently stated that the mosquito could reproduce in clean water compared to non-high school graduates (45% vs. 36.9%, *p* = 0.01). However, high school graduates less frequently stated that the mosquito could reproduce in rain water (4.1% vs 7.3%, *p* = 0.02). The latter was the only correct response on which the high school graduates had a significantly lower frequency than non-high school graduates (Table 1).

In relation to the availability of potential breeding sites, high school graduates more often mentioned tanks and sinks compared to non-high school graduates (Table 2). However, when asked about other containers, the high school graduates most frequently responded that they had no other container compared to non-high school graduates (11.7% vs. 6.8%, respectively, *p* = 0.01). It was interesting that most respondents with a sink (67.8%) wash it less than once per week. However, no differences were observed between high school graduates and non-graduates, in relation to the practices to prevent mosquitoes from breeding in these containers (Table 2).

**Table 2:** Availability of breeding sites and methods used to prevent mosquito reproduction

Dependent variables	Total	High school graduates	Non-high school graduates	*p* value
Do you have a tank for water?	186/1049 (17.7%)	126/613 (20.6%)	60/436 (13.8%)	0.01
Methods used to prevent mosquito breeding in the tank:				
Covers the tank	16/184 (8.7%)	12/125 (9.6%)	4/59 (6.8%)	0.53
Washes the tank	128/184 (69.6%)	86/125 (68.8%)	42/59 (71.2%)	0.74
Using temephos (Abate®)	14/184 (7.6%)	12/125 (9.6%)	2/59 (3.4%)	0.14
Uses bleach	11/184 (6%)	7/125 (5.6%)	4/59 (6.8%)	0.75
Other means	8/184 (4.3%)	8/125 (6.4%)	0/59 (0%)	0.05
None	26/184 (14.1%)	16/125 (12.8%)	10/59 (16.9%)	0.45
How do you wash the tank?:				
Just water	6/182 (3.3%)	3/122 (2.5%)	3/60 (5%)	0.37
Water and a brush	22/182 (12.1%)	16/122 (13.1%)	6/60 (10%)	0.54
With detergent	24/182 (13.2%)	14/122 (11.5%)	10/60 (16.7%)	0.33
With detergent and a brush	135/182 (74.2%)	92/122 (75.4%)	43/60 (71.7%)	0.59
With others	10/182 (5.5%)	8/122 (6.6%)	2/60 (3.3%)	0.37
Washes the tank less than once per week	40/182 (22%)	26/122 (21.3%)	14/60 (23.3%)	0.76
Has a sink to store water	567/1052 (53.9%)	358/614 (58.3%)	209/438 (47.7%)	< 0.001
Methods used to prevent mosquito breeding in the sink:				
Covers the sink	309/566 (54.6%)	195/357 (54.6%)	114/209 (54.5%)	0.99
Washes the sink	329/566 (58.1%)	204/357 (57.1%)	125/209 (59.8%)	0.54
Using temephos (Abate®)	37/566 (6.5%)	25/357 (7%)	12/209 (5.7%)	0.56
Uses bleach	46/566 (8.1%)	27/357 (7.6%)	19/209 (9.1%)	0.52
Other means	15/566 (2.7%)	8/357 (2.2%)	7/209 (3.3%)	0.43
None	35/566 (6.2%)	23/357 (6.4%)	12/209 (5.7%)	0.74
How do you wash the sink?:				
Just water	13/563 (2.3%)	8/356 (2.2%)	5/207 (2.4%)	0.90
Water and a brush	81/563 (14.4%)	48/356 (13.5%)	33/207 (15.9%)	0.42
With detergent	51/563 (9.1%)	34/356 (9.6%)	17/207 (8.2%)	0.59
With detergent and a brush	452/563 (80.3%)	282/356 (79.2%)	170/207 (82.1%)	0.40
With others	54/563 (9.6%)	39/356 (11%)	15/207 (7.2%)	0.15
Washes the sink less than once per week	376/555 (67.7%)	234/350 (66.9%)	142/205 (69.3%)	0.56
Other containers store water:				
Cans	182/1052 (17.3%)	107/614 (17.4%)	75/438 (17.1%)	0.90
Buckets	429/1052 (40.8%)	238/614 (38.8%)	191/438 (43.6%)	0.11
Jerrycan	92/1052 (8.7%)	48/614 (7.8%)	44/438 (10%)	0.21
Bottles	6/1052 (0.6%)	3/614 (0.5%)	3/438 (0.7%)	0.68
Pots	14/1052 (1.3%)	7/614 (1.1%)	7/438 (1.6%)	0.52
Other container	388/1052 (36.9%)	228/614 (37.1%)	160/438 (36.5%)	0.84
None - other	102/1052 (9.7%)	72/614 (11.7%)	30/438 (6.8%)	0.01

In relation to family and community attitudes and practices, high school graduates attend meetings (31.3% vs. 24.9%, *p* = 0.02) and stated that they educate members of their family and neighbors regarding measures to prevent dengue fever (46.6% vs. 37%, *p* = 0.002), more often than non-high school graduates (Table 3). In addition, high school graduates more often stated that they can carry out actions to control dengue (29.6% vs. 21.9%, *p* = 0.01) and that their suggestions to control dengue are heard (42% vs. 33.5%, p = 0.01). In relation to the difficulties involved in carrying out preventive measures against dengue, the high school graduates more often mentioned the lack of time (30.9% vs. 21.9%, *p* = 0.001) and, less often, the lack of resources (16.8% vs. 22.8%, p = 0.01), compared to those with lower levels of education (Table 3).

**Table 3:** Community attitudes and practices for dengue control

Dependent variables	Total	High school graduates	Non-high school graduates	*p* value
Attends community meetings	301/1052 (28.6%)	192/614 (31.3%)	109/438 (24.9%)	0.02
Participates in activities to prevent dengue in the neighborhood	200/1051 (19%)	124/613 (20.2%)	76/438 (17.4%)	0.24
Agrees with neighbors to collect garbage around the home	235/1052 (22.3%)	144/614 (23.5%)	91/438 (20.8%)	0.30
Do you educate other family members and/or neighbors regarding measures to prevent dengue?	447/1050 (42.6%)	285/612 (46.6%)	162/438 (37%)	0.002
Do you have the ability to take action to control dengue?	278/1052 (26.4%)	182/614 (29.6%)	96/438 (21.9%)	0.01
Do you consider your suggestions to control dengue are heard?	404/1051 (38.4%)	257/612 (42%)	147/439 (33.5%)	0.01
Do you lead campaigns to prevent dengue?	87/1052 (8.3%)	53/613 (8.6%)	34/439 (7.7%)	0.60
Do you get help for dengue prevention programs?	99/1048 (9.4%)	65/610 (10.7%)	34/438 (7.8%)	0.11
Do you file claims with the authorities when you consider they are not taking actions for dengue control in your community?	246/1049 (23.5%)	152/613 (24.8%)	94/436 (21.6%)	0.22
Reasons that hinder the implementation of preventive measures against dengue:				
Lack of time	286/1053 (27.2%)	190/614 (30.9%)	96/439 (21.9%)	0.001
Lack of information	592/1053 (56.2%)	349/614 (56.8%)	243/439 (55.4%)	0.63
Lack of funds	203/1053 (19.3%)	103/614 (16.8%)	100/439 (22.8%)	0.01
Other reason	79/1053 (7.5%)	44/614 (7.2%)	35/439 (8%)	0.62
You consider that the party responsible for taking measures to prevent dengue should be:				
The municipal government	590/1053 (56%)	335/614 (54.6%)	255/439 (58.1%)	0.26
Medical staff	62/1053 (5.9%)	37/614 (6%)	25/439 (5.7%)	0.82
Parents or guardians	118/1053 (11.2%)	68/614 (11.1%)	50/439 (11.4%)	0.87
Everyone / All people living in the community	490/1053 (46.5%)	297/614 (48.4%)	193/439 (44%)	0.16
Other	82/1053 (7.8%)	48/614 (7.8%)	34/439 (7.7%)	0.97

In the multiple regression models, after adjusting for age and sex, the high school graduates showed a higher frequency of correct answers in relation to knowledge about dengue symptoms and transmission (Table 4). However, high school graduates less frequently responded that the mosquito could reproduce in rain water (PR: 0.51; 95% CI: 0.3–0.88; *p* = 0.02). We hypothesized that this association could be explained because sewerage problems in the region would lead to the recognition of rainwater by the community as dirty and inadequate for the development of *Aedes spp*.

**Table 4:** Knowledge, practices and attitudes regarding dengue in relation to level of education - Models adjusted by demographic variables*

Dependent variables	Independent variables included in the models		
	High school	Age (every 10 years)	Female sex (vs. male)
Symptoms recognized as signs of dengue:			
Fever	1.06 (1.03–1.09)	NS	NS
Aches and pains	1.37 (1.14–1.66)	1.09 (1.02–1.16)	1.3 (1.02–1.65)
Red spots on the skin	1.55 (1.03–2.33)	NS	1.92 (1.07–3.45)
Knowledge regarding transmission:			
Knows how dengue is transmitted	1.17 (1.11–1.24)	NS	NS
Knows the name of the mosquito	2.69 (2.07–3.49)	1.16 (1.08–1.25)	NS
Knows what the mosquito looks like	1.66 (1.39–1.99)	1.11 (1.05–1.17)	NS
Means where the mosquito is believed to reproduce			
Clean water	1.44 (1.23–1.7)	1.15 (1.1–1.21)	1.26 (1.03–1.53)
Rainwater	0.51 (0.3–0.89)	NS	NS
Practices regarding dengue			
Does not store water in containers	2.13 (1.38–3.27)	1.19 (1.04–1.37)	NS
Attends community meetings	1.34 (1.08–1.66)	NS	NS
Do you educate other family members and/or neighbors regarding measures for prevention?	1.37 (1.17–1.6)	1.09 (1.04–1.14)	NS
Attitudes regarding dengue control.			
Do you have the ability to take action to control dengue?	1.55 (1.22–1.96)	1.13 (1.06–1.22)	NS
Do you consider your suggestions to control dengue are heard?	1.38 (1.17–1.64)	1.1 (1.04–1.16)	NS
Do you consider that lack of time hinders the implementation of preventive measures against dengue:	1.39 (1.11–1.74)	NS	NS

*For each of the dependent variables, a multiple regression model was obtained, including the variables of high school, age (as a continuous variable on the decade scale), and sex (female compared to male). This table only presents prevalence ratios and 95% confidence intervals of variables that were significant (*p* < 0.05). All these models were also adjusted by department (La Guajira vs others)

NS: non-significant (*p* > 0.05)

On the other hand, graduates showed a higher probability of practices that favor dengue control, including not storing water in containers, attend community meetings and educating family members and neighbors regarding prevention measures (Table 4). In addition, high school graduates showed more positive attitudes toward dengue control, such as considering that they have the ability to perform actions of control and that are heard when they make suggestions. However, high school graduates also stated the lack of time as a difficulty in carrying out preventive measures.

Age, which was included in the models as an adjustment variable, was significantly associated with correct answers on dengue knowledge, including the recognition of the symptom of aches and pains, the name and appearance of the mosquito, and knowing that it reproduces in clean water. In addition, with increasing age of participants, there was a greater frequency of preventive practices and positive attitudes, such as not storing water in containers and educating relatives and neighbors, as well as feeling that they have the ability to take action and that their suggestions are heard.

Finally, regardless of the educational level and age, women were more likely to recognize certain signs of dengue, such as aches and pains (PR: 1.3; 95% CI: 1.02–1.65; *p* = 0.03) and petechiae (PR: 1.92; 95% CI: 1.07–3.45; p = 0.03) and had a 26% greater probability of knowing that the mosquito reproduces in clean water (PR: 1.26; 95% CI: 1.03–1.53; *p* = 0.02), compared to men (Table 4).

## Discussion

This is a survey of important dimensions including more than 10 municipalities in the Caribbean region of Colombia. This, along with the probabilistic selection of participants would be strengths of this population survey. Based on these results, it can be inferred that almost the entire population studied recognizes dengue as a disease whose symptoms include fever. This suggests that this sign could be the potential motivation to turn to the health system in symptomatic cases. However, no other symptom is recognized by the majority of the population. In fact, each of the hemorrhagic manifestations was recognized as a sign of dengue in less than 15% of the respondents.

On the other hand, the majority of respondents stated that in order to care for dengue, they went to a doctor or a health institution. This suggests that this population recognizes dengue as a disease that requires medical attention. This could contrast with what has been observed in other populations that tend to self-medicate, even with antibiotics before consulting due to a fever [16].

In relation to level of education, the symptoms of fever, aches and pains and “red spots on the skin” (which we interpreted as petechiae), were recognized more often by high school graduates compared to non-high school graduates. Fever and aches and pains are very frequent signs in patients with dengue [17, 18]. In addition, skin manifestations, such as petechiae, can help us differentiate between dengue and other febrile diseases [19]. Therefore, it is plausible that people with a higher level of education would be better able to identify dengue and, therefore, seek professional help in time.

In turn, high school graduates had a higher frequency of correct responses to aspects related to transmission compared to non-high school graduates. This relationship between education and level of knowledge regarding dengue transmission and its control has already been observed in other studies [20, 21]. However, it is interesting that, in spite of this, there were no significant differences in relation to the frequency of domestic practices to prevent mosquito breeding sites. Moreover, in the population studied, knowledge about the transmission of dengue was not positively associated with intra-domiciliary control practices (data not shown). This suggests that knowledge related to the reproduction of the mosquito does not ensure the practice of vector control methods in their homes. This is consistent with what has been observed in other populations, in which the frequency of good practices of vector control tends to be lower than the level of knowledge [22, 23].

However, level of education was associated with practices at the community level, such as attending meetings, educating other members of the family and/or neighbors. Moreover, high school graduates more frequently felt they had the ability to take actions of control and that their suggestions were heard. This suggests that level of education could be related to the degree of empowerment of the population to integrate and combine efforts to control transmission. These results are consistent with those observed in other studies in which the level of education was shown to be an independent predictor of attitudes and practices regarding dengue [8, 24].

As additional findings, this study shows that regardless of the level of education, age is directly associated with certain knowledge regarding the disease and mechanisms of transmission. This association is consistent with what has been observed in other studies [24]. In addition, with age, an increase has been observed in the frequency of positive attitudes and practices to control transmission.

Finally, regardless of age and level of education, women have a higher frequency of correct responses in relation to questions about knowledge regarding the disease and transmission. This could suggest that women may be more interested in or sensitive to acquiring skills that allow them to recognize the disease and prevent its transmission.

The limitations of this study include the difficulty of measuring how closely the answers about practices and attitudes, referred to by respondents, are related to those adopted in daily life. However, the results of this study provide evidence of how factors such as level of education and demographics (i.e. age and sex) could be determining factors of knowledge regarding the disease and its transmission, as well as attitudes and practices, especially those that involve the integration of community efforts to control the burden associated with dengue.

It is important to consider that the questionnaire was administered to the adult who was at home at the time of the visit, who were mostly women. This selection strategy could cause a selection bias that affects the representativeness of the survey. However, it is to be assumed that the person surveyed had a prominent role in the activities carried out at home. Therefore, we expected that the sample interviewed would adequately represent the control practices of those communities. In addition, after adjusting the demographic variables, we found that the associations were independent of variables such as sex and age.

On the other hand, compared to self-administered questionnaires, those administered by health personnel could promote socially desirable responses. The standardization of the questionnaire and the training of the interviewer could have minimized the risk of this information bias. On the other hand, if that bias occurred, it was probably not differential. Therefore, we believe that the associations found may persist regardless of the questionnaire administration strategy.

In Colombia, the health authorities lead the National Integrated Management Strategy for the Prevention and Control of Dengue that seeks to reduce morbidity and associated lethality. This strategy includes a component of social promotion, which comprises a regular program of permanent activities that includes the Communication for behavioral impact (COMBI) strategy [25]. However, one of the main challenges of this strategy is the continuity of community participation [26]. In order to guarantee the sustainability of these strategies, it is necessary the continuous monitoring of the community and the recognition of the behavior determinants.

In dengue, as well as for other tropical diseases, education is a fundamental pillar for integrated control, prevention and promotion. This determinant should be considered in the development of public policies that may be able to steadily reduce the disease and its impact on endemic regions. Specifically, the results of this study suggest that educational campaigns could focus on people with low educational levels, taking into account age groups. These targeted interventions could be more efficient and have a greater impact on preventive attitudes and practices.

## Conclusions

Level of education could be a key determinant of knowledge of the disease and its transmission, as well as attitudes and practices, especially those that involve the integration of community efforts for dengue control. The results suggested that populations with a low educational level are especially vulnerable and their integration in control programs could be particularly difficult. Therefore, prioritizing these populations in programs aimed at controlling dengue and other arboviruses would be justified.

## Abbreviations

95% CI: 95% Confidence Interval
KAPs: Knowledge, attitudes and practices
PR: Prevalence Ratio
